# The efficacy and safety of epinephrine for postoperative bleeding in total joint arthroplasty: A PRISMA-compliant meta-analysis

**DOI:** 10.1097/MD.0000000000006763

**Published:** 2017-04-28

**Authors:** Yanbin Teng, Jianxiong Ma, Xinlong Ma, Ying Wang, Bin Lu, Chaowei Guo

**Affiliations:** Biomechanics Labs of Orthopaedics Institute, Tianjin Hospital, Tianjin, PR China.

**Keywords:** adrenaline, epinephrine, meta-analysis, postoperative bleeding, total hip arthroplasty, total joint arthroplasty, total knee arthroplasty

## Abstract

**Background:**

Total joint arthroplasty (TJA) usually results in postoperative bleeding. Some randomized controlled trials (RCTs) and nonrandomized controlled trials (non-RCTs) have been performed to evaluate the effects of epinephrine on postoperative bleeding after TJA. However, this remained controversial about the efficacy and safety of epinephrine for postoperative bleeding in TJA. The objective of our meta-analysis was to compare the overall effect and safety of epinephrine and placebo for postoperative bleeding in TJA.

**Methods:**

PubMed, Embase, and the Cochrane Library were searched to identify potentially relevant articles. RCTs or non-RCTs involving epinephrine and placebo for blood loss in total knee arthroplasty or total hip arthroplasty were included. Our study was performed based on the Preferred Reporting Items for Systematic Reviews and Meta-Analyses statement. RevMan v5.3 was used to analyze the relevant data.

**Results:**

Four RCTs and 1 non-RCT involving 646 participants met the inclusion criteria. The overall pooled results from meta-analysis demonstrated that compared with control groups, epinephrine groups could significantly reduce the postoperative bleeding volume (mean difference [MD] = −168.42, 95% confidence interval [CI]: −272.37 to −64.47, *P* = 0.001). There was no significant difference in intraoperative bleeding volume between epinephrine and control groups (MD = −12.89, 95% CI: −53.45 to 27.69, *P* = 0.53). No significant difference was found between 2 groups in terms of postoperative hemoglobin loss (MD = −0.28, 95% CI: −0.66 to 0.10, *P* = 0.15). Compared with the control groups, no statistically significant difference was found in terms of postoperative transfusion rate in epinephrine groups (relative risk [RR] 0.86, 95% CI: 0.64–1.15, *P* = 0.31). In addition, the results of the meta-analysis also indicated no significant difference in terms of the incidence rate of deep venous thrombosis (DVT) between 2 groups (RR 0.28, 95% CI: 0.05–1.64, *P* = 0.16).

**Conclusion:**

The meta-analysis showed that epinephrine could significantly reduce postoperative bleeding volume in TJA without increasing the incidence of DVT. However, there was no significant reduction in intraoperative bleeding volume, postoperative hemoglobin loss, and transfusion rate after the administration of epinephrine.

**Limitations:**

In this study, a higher heterogeneity and a risk of selection bias may be present in postoperative hemoglobin loss. In addition, the sample size of the included studies was too small, so our findings need to be further validated with more high-quality and larger scale RCTs in the future.

**Systematic review registration number:**

None.

## 1 Introduction

Reduced postoperative bleeding in patients is an important part of perioperative treatment. Total hip arthroplasty (THA) or total knee arthroplasty (TKA) in patients with postoperative bleeding, to a certain extent, will increase the chances of allogeneic blood transfusion. Despite the fact that tranexamic acid has been extensively studied for reducing postoperative bleeding in patients undergoing total joint arthroplasty (TJA), there has been little research into the role of epinephrine in reducing postoperative bleeding. The procoagulant effect of epinephrine is achieved mainly by the following 2 aspects: first, it reduces platelet transport time in the spleen and increases platelet aggregation by α-adrenergic activation, resulting in an instant increase in platelet count by 20% to 30%; second, epinephrine can promote the release of multiple coagulation factors through β-adrenergic activation, such as fibrinogen. Because epinephrine is often used in conjunction with tranexamic acid, it is necessary to study the efficacy and safety of epinephrine in reducing postoperative bleeding in patients undergoing TJA.

Postoperative bleeding is an inevitable complication of TJA. Complications of the circulatory system and high blood transfusion rate are closely related to a large number of postoperative bleeding. Approximately half of the patients who underwent total hip or TKA received more than 2 U of blood transfusion after surgery.^[1,2]^ Allogeneic blood transfusion after TJA is very common, but allogeneic blood transfusion is not without risk. This risk includes immune-related transfusion reactions such as nonhemolytic transfusion febrile reactions, allergic reactions, hemolytic reactions, transfusion-related acute lung injury, transfusion-related graft versus host disease, and immunosuppression, and also immunosuppressive transfusion reactions, such as bacterial contamination response, circulating overload, the impact of transfusion on the liver, and disease transmission. Therefore, it is necessary to choose a safe and effective measure to reduce postoperative bleeding and reduce blood transfusion after TJA surgery.

In the past few years, a number of randomized controlled studies have been performed on epinephrine for postoperative bleeding after TJA.^[3–7]^ Although these studies have led to some conclusions, the effect of epinephrine on postoperative bleeding in the TJA does not exist in meta-analysis or systematic review. The aim of our study was to examine the effectiveness and safety of epinephrine in reducing postoperative bleeding after TJA in order to better serve the clinical work.

## 2 Methods

Our research strictly complies with Preferred Reporting Items for Systematic Reviews and Meta-analyses principles. Since this was a meta-analysis of previously published studies, no patient-informed consent and ethical approval were required. Our literature search was performed in the following databases from 1975 to 2016: PubMed, Embase, and Cochrane. The following keywords including postoperative bleeding, postoperative blood loss, total hip arthroplasty, total hip replacement, total knee arthroplasty, total knee replacement, epinephrine, and adrenaline were used for searching.

### 2.1 Inclusion criteria

The included studies need to meet the following criteria—population: patients with unilateral primary TJA. Intervention group: epinephrine. Control group: saline or placebo. Outcomes: at least 1 of the following items appeared—intraoperative bleeding volume, postoperative bleeding volume, postoperative transfusion rate, postoperative hemoglobin loss, and serious side effects of treatment. Study design: randomized controlled trials (RCTs) or case–control trials were published in English.

### 2.2 Exclusive criteria

Patients were excluded from the meta-analysis if they had bilateral TJA, neoplastic disease, traumatic fracture, mental illness, and prosthetic implant rejection.

### 2.3 Selection criteria

The literature screening was done by 2 reviewers independently of each other according to the above inclusion and exclusion criteria. Whether the literature was included in the decision by the 2 reviewers, and if there was disagreement, the disagreement was resolved through discussion or the judgment of the third person. The qualities of all included RCTs were based on Cochrane collaboration tool.^[8]^ The evaluation standards were included as follows: randomization generation method; allocation concealment; blinding of participant, personnel, and assessor; selective reporting; and other biases. The qualities of nonrandomized controlled trials (non-RCTs) were based on MINORS quality assessments with scores ranging 0 to 24.^[9]^

### 2.4 Data extraction

Data from the enrolled literatures were extracted by 2 reviewers independently. The extracted data were included as follows: the first author's name, publication data, the size of sample, epinephrine administration route, epinephrine dose, postoperative bleeding volume, transfusion rate, and the incidence rate of deep venous thrombosis (DVT).

### 2.5 Statistical analysis

The results are calculated by RevMan 5.3 (The Cochrane Collaboration, Oxford, United Kingdom). Statistical heterogeneity is tested by the standard chi-square test depending on the value of *P* and *I*^2^. When no significant heterogeneity is found (*P* > 0.10 or *I*^2^ < 50%), a fixed effect model is chosen; otherwise, a random effect model is adopted. Continuous outcomes are expressed as the standardized mean difference (MD) with 95% confidence intervals (CIs) such as intraoperative bleeding volume, postoperative bleeding volume, and postoperative hemoglobin loss. Discontinuous outcome is expressed as relative risk (RR) with 95% CIs such as transfusion rate and the incidence of DVT. *P* < 0.05 is considered to be statistically significant for the differences in means. Sensitivity analysis was used to evaluate the stability of the results epinephrine versus placebo for postoperative bleeding in TJA. We conducted sensitivity analysis to explore the source of heterogeneity by omitting the study sequentially.

## 3 Results

### 3.1 Literature search

**Figure 1:**
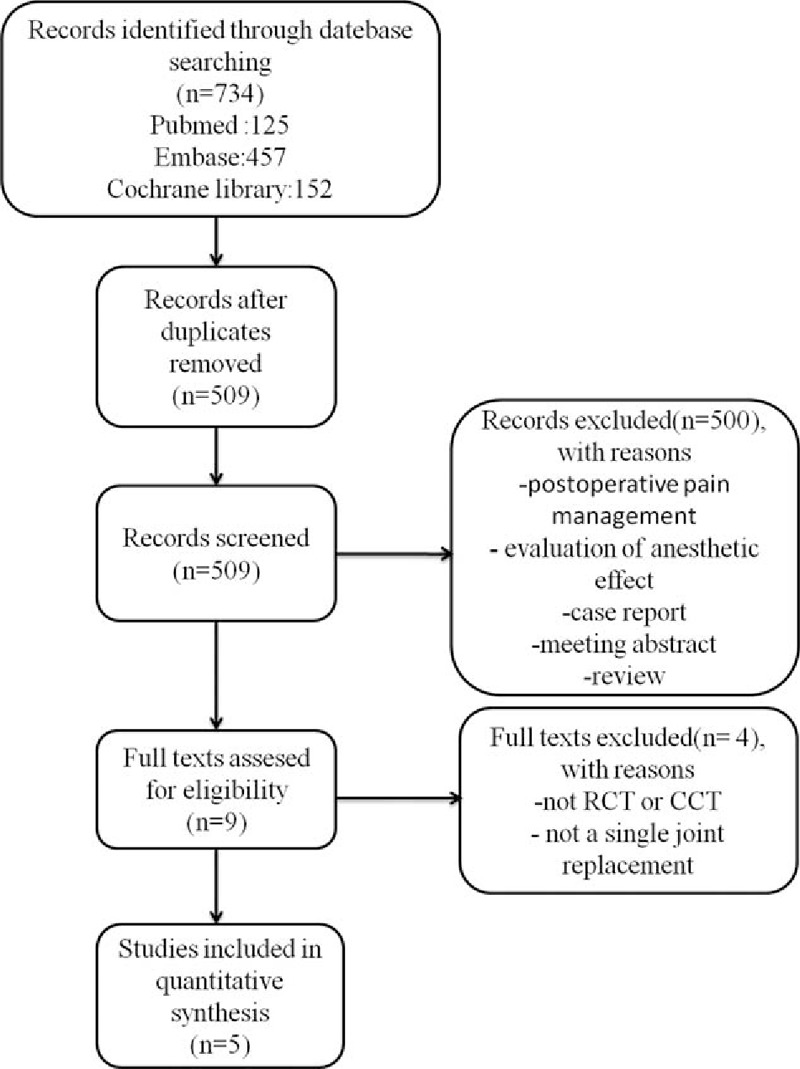
The selection of literature for included studies.

A total of 734 potential studies were identified with the first search strategy. Of these, 225 literatures had been identified as duplicated studies and were excluded. Then, 504 articles were excluded by screening the titles and reading the abstracts and the full texts, as they did not meet the inclusion criteria. Eventually, only 5 papers were included in the meta-analysis.^[3–7]^ These studies included a total of 335 patients in the epinephrine group and 311 patients in the control group. More details of the literature search are shown in Fig. 1.

### 3.2 Study characteristics

The characteristics of the 5 included studies are presented in Table 1. Malone(D)2009 and Malone(P)2009 were from the same literature,^[7]^ which included 2 research groups, and they were performed by 2 surgeons, respectively; therefore, we separated the study performed by the 2 doctors. In 4 studies, epinephrine was administered by local infiltration,^[3,5–7]^ but in 1 study, it was administered by intravenous injection.^[4]^ Although the amounts of epinephrine are not the same, they are all low concentrations. The mean age in epinephrine group ranges from 58.6 to 72.08 and control group ranging from 61.7 to 69. There are 3 articles on THA,^[3,4,6]^ and 2 papers on TKA.^[5,7]^ Among the 5 papers, France, the United States, and Denmark each had 1 literature,^[3,4,7]^ while the other 2 were from China.^[5,6]^ Other baseline characteristics are very similar between the epinephrine and control groups.

**Table 1:**
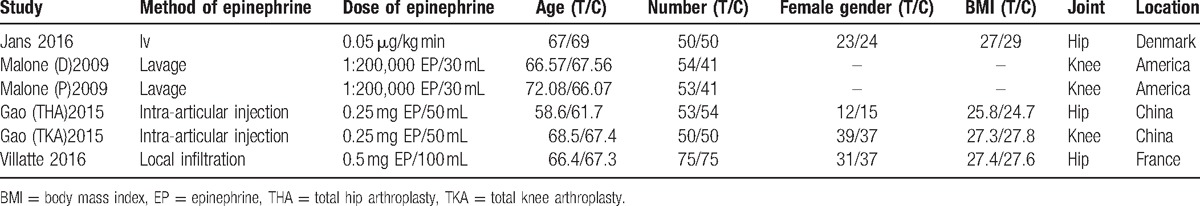
The characteristics of the included epinephrine studies.

### 3.3 Risk of bias assessment

**Figure 2:**
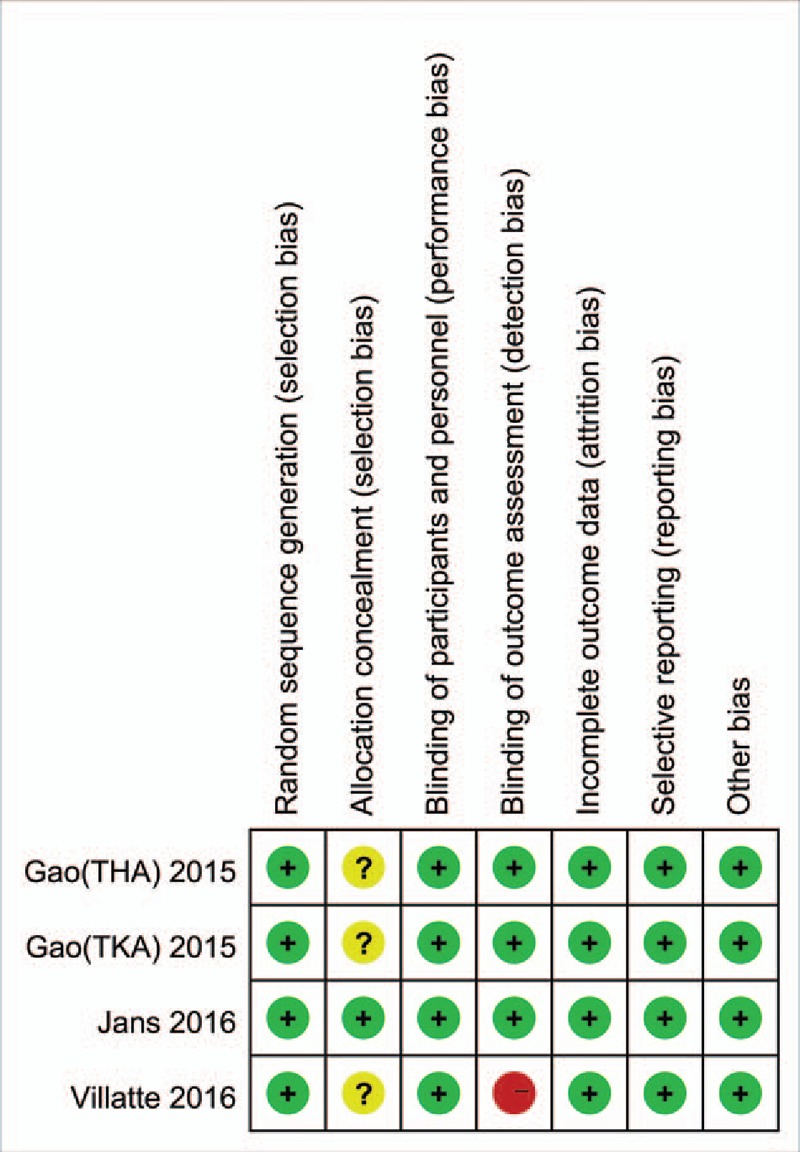
The summary of bias risk of randomized controlled trials.

The Cochrane collaboration tool was used for the assessment of the RCTs, all the included RCTs showed a low risk of bias (Fig. 2). The MINORS quality assessment used for the assessment of the only non-RCT study is presented in Table 2.

**Table 2:**
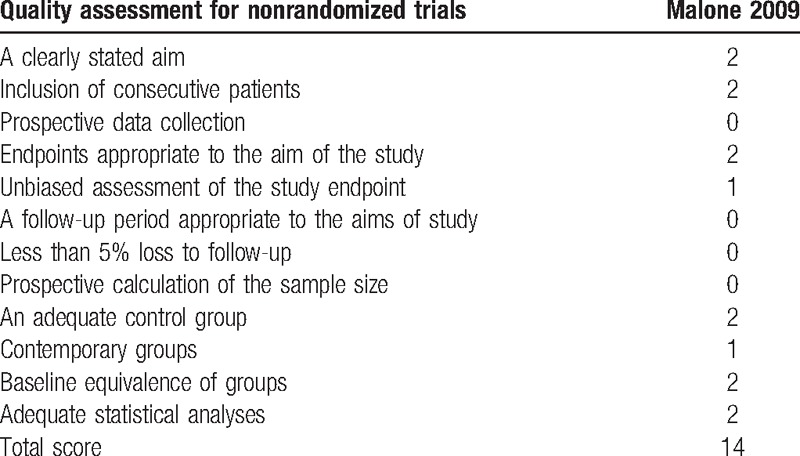
The MINORS quality assessment of nonrandomized controlled trial.

### 3.4 Outcomes for meta-analysis

#### 3.4.1 Intraoperative bleeding volume

**Figure 3:**
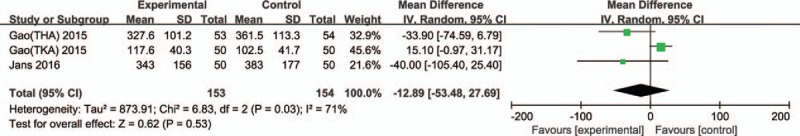
Forest plot of intraoperative bleeding volume between 2 groups.

Details regarding intraoperative bleeding volume were available in 3 trials.^[4–6]^ There was significant heterogeneity (χ^2^ = 6.83, df = 2, *I*^2^ = 71%, *P* = 0.03); therefore, a random effect model was performed. No statistically significant difference in intraoperative bleeding volume was found between epinephrine and control groups (MD = −12.89, 95% CI: −53.45 to 27.69, *P* = 0.53; Fig. 3).

#### 3.4.2 Postoperative bleeding volume

**Figure 4:**
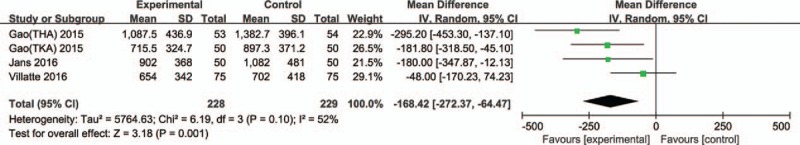
Forest plot of postoperative bleeding volume between 2 groups.

Details regarding postoperative bleeding volume were available in 4 trials.^[3–6]^ There was significant heterogeneity (χ^2^ = 6.19, df = 3, *I*^2^ = 52%, *P* = 0.10); therefore, a random effect model was applied. The overall pooled results from meta-analysis demonstrated that compared with control groups, epinephrine groups could significantly reduce the postoperative bleeding volume (MD = –168.42, 95% CI: −272.37 to −64.47, *P* = 0.001; Fig. 4).

#### 3.4.3 Postoperative hemoglobin loss

**Figure 5:**
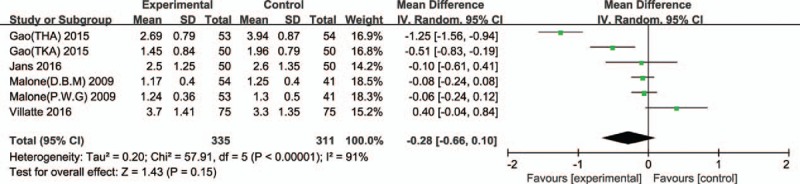
Forest plot of postoperative hemoglobin loss between 2 groups.

Six trials reported postoperative hemoglobin loss in TJA.^[3–7]^ Significant heterogeneity was found, so a random model was applied (χ^2^ = 57.91, df = 5, *I*^2^ = 91%, *P* < 0.00001). No significant difference in postoperative hemoglobin loss was found between epinephrine and control groups (MD = −0.28, 95% CI: −0.66 to 0.10, *P* = 0.15; Fig. 5).

#### 3.4.4 Postoperative transfusion rate

**Figure 6:**
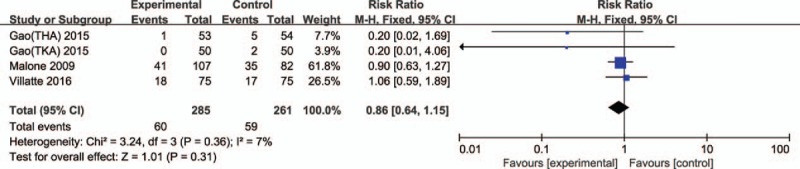
Forest plot of postoperative transfusion rates between 2 groups.

Four trials reported postoperative transfusion rate in TJA. No significant heterogeneity was found, so a fixed effect model was applied (χ^2^ = 3.24, df = 3, *I*^2^ = 7%, *P* = 0.36). Compared with the control groups, no significant difference was found in epinephrine groups (RR 0.86, 95% CI: 0.64–1.15, *P* = 0.31; Fig. 6).

#### 3.4.5 Serious side effects of treatment

**Figure 7:**
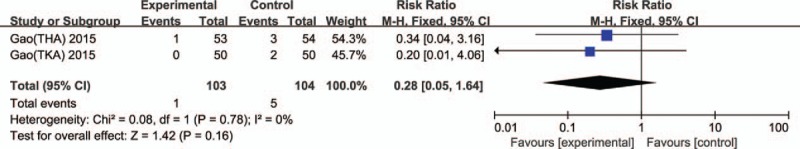
Forest plot of the incidence rates of deep venous thrombosis between 2 groups.

DVT was the most commonly reported serious side effect of treatment in the trials included in our study. Two studies reported the incidence rate of DVT. Significant heterogeneity was not found; therefore, a fixed effect model was performed (χ^2^ = 0.08, df = 1, *I*^2^ = 0%, *P* = 0.78). The results of the meta-analysis indicated no significant difference in terms of the incidence rate of DVT between epinephrine and control groups (RR 0.28, 95% CI: 0.05–1.64, *P* = 0.16; Fig. 7).

### 3.5 Sensitivity analysis

We found that the results of heterogeneity comparing postoperative hemoglobin loss were not significantly reduced by omitting the study sequentially. However, the results of heterogeneity comparing intraoperative bleeding volume significantly reduced (MD = −35.60, 95% CI: −70.15 to −1.06, *P* = 0.88, *I*^2^ = 0%) after excluding the Gao (TKA) et al^[5]^ study. Therefore, the Gao (TKA) et al^[5]^ study was regarded as the source of heterogeneity. Similarly, the Villatte et al^[3]^ study was also considered as the source of heterogeneity since the heterogeneity significantly reduced after excluding the literature in the total results comparing postoperative bleeding volume (MD = −216.47, 95% CI: −304.51 to −128.43, *P* = 0.50, *I*^2^ = 0%).

## 4 Discussion

As far as we know, this meta-analysis first reviewed the relevant literatures systematically and compared the efficacy and safety of epinephrine versus placebo for postoperative bleeding in TJA. The data analysis showed that there was no significant reduction in intraoperative bleeding volume between epinephrine and control groups. This finding of the meta-analysis is consistent with previous studies.^[10,11]^ The result may be related to the time of epinephrine administration and methods of applying epinephrine during the procedure. Although the platelets of the spleen are immediately released after the administration of epinephrine, the peak of the coagulation factor cannot be reached until more than 20 minutes after administration.^[12]^ Thus, the relatively short operation time of TJA may affect the effects of epinephrine. The mean value of intraoperative bleeding volume of Gao (TKA) study was significantly different from that of the other 2 studies, and it was only about 1/3 of the values from 2 other studies. Jans et al^[4]^ and Gao (THA) et al^[6]^ studied the patients with THA, while Gao (TKA) et al^[5]^ studied the subject of TKA. The reason for this difference may come mainly from the following 2 aspects. First, the application of tourniquet: tourniquets are often used in TKA, but they are not used in THA. This may be the main reason. Second, the amount of soft tissue: because the hip soft tissue is more abundant than the knee, so the amount of intraoperative blood loss in THA may be more.

Postoperative bleeding volume is the main evaluation criteria of the efficacy of epinephrine. Fortunately, compared with control groups, epinephrine groups could significantly reduce the postoperative bleeding volume in our study. This is consistent with the role of epinephrine in promoting platelet aggregation and several coagulation factor release.^[12,13]^ Although low doses of epinephrine can cause beta-adrenergic activation, resulting in decreased peripheral vascular resistance and increased circulating blood volume, this effect disappears within 5 minutes of cessation of epinephrine administration.^[13]^ Therefore, this hemodynamic effect did not significantly affect the procoagulant and hemostatic effects of epinephrine. This finding is in accordance with the former studies about the effect of epinephrine in various operations.^[14,15]^

Postoperative hemoglobin loss is also a vital indicator to evaluate the efficacy of epinephrine for postoperative bleeding. However, no significant reduction in postoperative hemoglobin loss was found in the epinephrine group compared with control group. This could be explained by the ability to regenerate hemoglobin, the level of stored hemoglobin, and the small sample size.

The transfusion rate can indirectly reflect the efficacy of epinephrine. Unfortunately, compared with the control group, no significant difference in transfusion rate was found in epinephrine groups. This may be due to a lack of uniform blood transfusion criteria, and blood transfusion decisions were often based on a combination of hemoglobin levels and patient's subjective feelings. In addition, it was closely related to the amount of blood reserves. Patients might have been transfused more liberally if the blood reserves were adequate; conversely, patients may have been transfused more restrictively. At the same time, because the number of literatures about this study was very limited and the trials were conducted in only a few countries, this led the patient population to be too single and lack heterogeneity. This is also one of the limitations of our research, so we need more studies with larger sample sizes.

Any interventions associated with procoagulants to reduce bleeding must be examined carefully for the risk of venous thrombosis events, especially DVT. The incidence rate of DVT is a vital indicator to evaluate the safety of epinephrine for postoperative bleeding. It was worth mentioning that there was no statistical difference in the incidence rate of DVT between epinephrine and control groups. Although the current finding suggests that epinephrine does not increase the incidence of DVT, we also need more relevant studies to support this conclusion in the future. However, there were also several other side effects reported in the included literature, including dizziness, bradycardia, superficial wound infections, and hematoma.^[4–6]^ Several other complications have been reported in some studies, such as increased skin lesion development, delayed wound healing, skin margin necrosis, and arthrofibrosis.^[14–16]^

In our meta-analysis, epinephrine was first evaluated in TJA in terms of intraoperative bleeding volume, postoperative bleeding volume, postoperative transfusion rate, postoperative hemoglobin loss, and incidence of DVT. However, previous meta-analyses were mostly concerned on the efficacy and safety of tranexamic acid for postoperative bleeding in THA or TKA.^[17,18]^ Our findings were consistent with several other studies, by Gasparini et al^[14]^ and Anderson et al,^[15]^ which have shown that epinephrine can effectively decrease postoperative blood loss in TKA. On the contrary, some other literatures reported that epinephrine could not effectively reduce postoperative bleeding in TJA.^[7,19]^ Nevertheless, the statistics of postoperative bleeding volume may be different in these studies, the researchers may not have taken this part of the hidden bleeding into account, and it may be inaccurate to calculate the amount of postoperative blood loss through the change of perioperative hemoglobin. In addition, epinephrine reduces bleeding during perioperative periods by contracting peripheral vessels^[14]^ and further reduces bleeding through the hemostatic effect of platelets activated by α_2_-adrenergic after surgery.^[20]^ Because the antifibrinolytic effect of tranexamic acid can synergize with the vasoconstriction effect of epinephrine, their combined use can enhance the effective time of each other, so it is equally meaningful to study the efficacy of epinephrine for postoperative bleeding. There were 3 literatures that reported the use of tranexamic acid, Jans et al,^[4]^ Gao et al. (TKA),^[5]^ and Gao (THA) et al,^[6]^ respectively. However, in the above studies, the epinephrine and the control groups were added the same little amount of tranexamic acid, so the variant was controllable.

Our research has relatively high heterogeneity, which may mainly come from the following aspects: first, only 5 papers were included, including 4 RCTs and 1 non-RCT literature, and the overall quality of the included literature was low and the available data were very limited. Second, there were some differences in the joints that needed to be replaced—surgical approach, incision technique, type of prosthesis, and method of fixation. Third, there were some differences in the method of calculating the amount of blood loss, and some of the included literature did not calculate hidden blood loss.^[3,7]^ In general, all of the above points may affect the heterogeneity of our study.

There are several notable limitations in our study. First, the sample size of the included studies was too small, which reduced the level of evidence for this study. Second, the follow-up time was not clear in some studies, and the long-term effects of epinephrine were not evaluated due to lack of long-term follow-up. Third, the optimal dose and route of administration cannot be clearly defined in our study. Fourth, some other side effects such as arrhythmias and psychiatric symptoms cannot be evaluated due to lack of data. Therefore, these findings need to be further validated with more high-quality and larger scale RCTs in the future.

## 5 Conclusion

The results of our meta-analysis showed that epinephrine significantly reduced postoperative bleeding volume in TJA without increasing the incidence of DVT. However, epinephrine did not significantly reduce intraoperative bleeding volume, postoperative hemoglobin loss, and transfusion rate compared with the control groups in patients undergoing TJA.
